# SMURF2 facilitates ubiquitin-mediated degradation of ID2 to attenuate lung cancer cell proliferation

**DOI:** 10.7150/ijbs.80979

**Published:** 2023-06-26

**Authors:** Mingwei Han, Yixiao Guo, Yiming Li, Qingmei Zeng, Wanwan Zhu, Jianli Jiang

**Affiliations:** National Translational Science Center for Molecular Medicine and Department of Cell Biology, Fourth Military Medical University, Xi'an 710032, Shaanxi, China

**Keywords:** non-small cell lung cancer, SMURF2, post-translational modifications, cell cycle, tumor progression

## Abstract

SMAD-specific E3 ubiquitin protein ligase 2 (SMURF2) functions as either a tumor promoter or tumor suppressor in several tumors. However, the detailed effect of SMURF2 on non-small cell lung cancer has not been fully understood. In this study, SMURF2 expression and its diagnostic value were analyzed. Co-Immunoprecipitation (Co-IP), proximity ligation assay (PLA), chromatin immunoprecipitation (ChIP) and nude mice tumor-bearing model were applied to further clarify the role of SMURF2 in lung cancer. SMURF2 expression was reduced in the tumor tissues of patients with NSCLC and high SMURF2 expression was significantly correlated with favorable outcomes. Furthermore, the overexpression of SMURF2 significantly inhibited lung cancer cell progression. Mechanistically, SMURF2 interacted with inhibitor of DNA binding 2 (ID2), subsequently promoting the poly-ubiquitination and degradation of ID2 through the ubiquitin-proteasome pathway. Downregulated ID2 in lung cells dissociates endogenous transcription factor E2A, a positive regulator of the cyclin-dependent kinase inhibitor p21, and finally induces G1/S arrest in lung cancer cells. This study revealed that the manipulation of ID2 via SMURF2 may control tumor progression and contribute to the development of novel targeted antitumor drugs.

## Introduction

Lung cancer remains the leading cause of cancer-related death worldwide 1. Although studies have shown that five-year survival for patients with lung cancer in the US has improved over the past decade, the incidence has increased by 12.16% in China 2. More studies are needed to investigate biomarker-based diagnosis, treatment, and drug development 3. Typically, the loss of cell cycle regulation is a common event in NSCLC, and multiple cell cycle regulatory proteins play key roles in tumorigenesis. The clinical use of cyclin-dependent kinase (CDK) inhibitors has resulted in favorable outcomes for patients with tumors 4. Therefore, it is important to elucidate the mechanism of cell cycle dysregulation and to develop targeted therapeutic agents for patients with NSCLC.

Cell cycle progression in eukaryotes integrates efficient and rapid regulation of multiple transcription factors. Cell cycle regulation in NSCLC involves a variety of molecular and protein interactions in several signaling pathways 5. Molecules involved in cell cycle regulation undergo various post-translational modifications (PTM), among which ubiquitination is highly conserved and fundamental in regulating the physiological levels and activity of proteins 6. SMAD specific E3 ubiquitin protein ligase 2 (SMURF2) was initially identified as an HECT-type E3 ubiquitin ligase that negatively regulates the transforming growth factor beta (TGF-beta) signaling 7. In recent years, the biological roles of SMURF2 have been identified in different tumors, including tumor metastasis, apoptosis, cell cycle progression, senescence, and genomic stability 8. Studies on Smurf2^-/-^ mice have indicated that increased age leads to the spontaneous development of various tumors, including liver, blood, lung, pituitary, and Harderian gland tumors 8, 9. In senescent cells, SMURF2 mediates the ubiquitination of inhibitor of differentiation 1 (ID1) and subsequently regulates p16 expression 10. In physiological state, SMURF2 is mainly aggregated and localized in the nucleus, and degrades several nuclear proteins 11. Nevertheless, further analysis of the function of SMUFR2 and its role in tumorigenesis is required.

ID2 belongs to the helix-loop-helix (HLH) transcription factor (TF) family and promotes tumor progression, enhances cell proliferation, and inhibits the activity of basic helix-loop-helix (bHLH) transcription factors 12, 13. In Ewing sarcoma tumors, ID2 was found to be a critical regulator of developmental-related genes and tumor growth both *in vivo* and *in vitro* 14. Moreover, elevated ID2 levels are closely related to the poor prognosis of patients with breast cancer and colonization of breast cancer cells in the brain 15, 16. Additionally, overexpression of ID2 has been identified in colorectal cancer (CRC), and KD of ID2 leads to decreased proliferation and cell cycle arrest in CRC cell lines, accompanied by significantly altered expression levels of a variety of cell cycle-related proteins, including cyclin D1, cyclin E, Cdk6, p21, and p27 17. ID2 has been identified as a dynamic and unstable protein during cell cycle transition, and when cells enter the quiescent stage, ID2 is eliminated. However, the targeted degradation mechanism of this process has not been elucidated.

Notably, the biological function of ID2 in lung cancer remains unclear. Bioinformatics analysis of ID family members showed that elevated mRNA expression of ID2 was associated with improved overall survival 18. Another study found that the expression of ID2 and PD-1 was positively correlated in patients with lung squamous cell carcinoma 19. ID2 appears to be decreased in patients with lung cancer. Moreover, ID2 has also been reported to regulate squamous cell carcinoma proliferation through the NF-κB/cyclin D1 pathway 20. Additionally, studies have reported that Med1 deletion promotes the invasion and metastasis of human NSCLC cells by upregulating ID2 and other metastasis-related genes 21. Immunohistochemical staining of clinical samples from patients with NSCLC showed that the localization and expression of intracellular ID2 was valuable for the prognosis of patients. Only the nuclear expression of ID2 was negatively correlated with tumor grade 22. High expression of ID2 in the nucleus is an independent and unfavorable factor for the prognosis of patients with tumors 22. In conclusion, all these studies provide more detailed insights into the involvement of ID2 in the regulation of cell cycle progression and provide a novel therapeutic approach for targeting the ID protein family. Given the complexity of the role of ID2 in lung cancer, it is necessary to conduct in-depth research on its detailed mechanisms.

Here, we demonstrate that ID2 degradation by the E3 ubiquitin ligase SMURF2 through ubiquitin-proteasome system plays a critical role in regulating the cell cycle. Downregulation of ID2 significantly increased p21 expression and inhibited cell cycle progression via E2A-mediated transcriptional activation of cyclin-dependent kinase-inhibitor 1 (CDKN1A). However, overexpression of ID2 reduced G1/S arrest in the lung cells. Our results provide new insights into the rapid regulation of ID2 protein levels and cell cycle progression by E3 ubiquitin ligase. Further research may provide more information for the development of targeted therapeutic drugs.

## Materials and methods

### Cell lines and culture

A549 cells were obtained from the American Type Culture Collection (ATCC-CCL-185; Manassas, VA, USA). H460, H1650, H1975, and HEK293T cells were obtained from the Cell Bank of the Chinese Academy of Sciences (Shanghai, China). RPMI1640 (10-040-CVRC; Corning, NY, USA) and DMEM supplemented with 10% fetal bovine serum (10100-147; Gibco, China), 1% penicillin/streptomycin, and 2% glutamine were used for cell culture. The cells were maintained at 37°C in 5% CO_2_ and were routinely tested for mycoplasma. All cell lines in this study were tested and authenticated using short tandem repeat DNA profiling by the Beijing Microread Genetics Co., Ltd. (Beijing, China).

### Antibodies and Reagents

Cycloheximide (CHX) and MG132 were purchased from MCE (HY-12320; MedChemExpress, China) and Selleck (S2619, China), respectively. The antibodies used in this study were as follows: anti-β-actin (M1210-2; HUABio, Hangzhou, China), anti-SMURF2 (12024S; CST, Danvers, Massachusetts, USA; bs-4056R; Bioss, Beijing, China), anti-ID2 (ab166708; Abcam, China; 3431S; CST; MA5-32891; Invitrogen, USA), anti-E2A (ab228699; Abcam), anti-Flag (14793S; CST), anti-GFP (OSE00003G; Invitrogen), anti-HA (3724T; CST), anti-Rabit IgG (3900S; CST), anti-p21 (10355-1-ap; Proteintech, Wuhan, Hubei, China; ab107099; Abcam; 2947T; CST), anti-Ubiquitin (3936T; CST).

### Plasmid constructions

Open reading frames encoding N-terminal Flag-tagged SMURF2 and its four truncated plasmids were generated using pcDNA3-Flag-ASC templates (GeneChem, Shanghai, China). Plasmids encoding full-length human ID2 (GeneChem) were cloned into a GFP-tagged target vector, and ubiquitin (GeneChem) was cloned into an HA-tagged target vector for immunoprecipitation or immunoblotting. Full-length HLH domain-mutated ID2 (ID2^δHLD^) was tagged with GFP (GeneChem). The blank vector expressing only tag protein was used as control and referred to as 'EV'.

### Gene KD and overexpression

Lentiviruses containing shRNAs targeting human SMURF2 were obtained from GeneChem. Cells were seeded 12 h prior to infection, and to establish stable cell lines, cells were selected using puromycin (2 μg/mL) for at least two weeks. Gene overexpression was achieved by transfecting cells with the corresponding plasmids, and the cells were plated in six-well plates at 1 × 10^6^ cells/well. Three siRNAs targeting ID2 were designed and synthesized (Beijing Tsingke Biotech Co., Ltd.) with the following sequences:

siID2-1 (Sense): 5′-CGAUGAGCCUGCUAUACAA-3′,

siID2-1 (Anti-sense): 5′-UUGUAUAGCAGGCUCAUCG-3′;

siID2-2 (Sense): 5′-GACUGCUACUCCAAGCUCA-3′,

siID2-2 (Anti-sense): 5′-UGAGCUUGGAGUAGCAGUC-3′;

siID2-3 (Sense): 5′-CUUCUGAGUUAAUGUCAAA-3′,

siID2-3 (Anti-sense): 5′-UUUGACAUUAACUCAGAAG-3′.

All transfection procedures were performed using Lipofectamine 2000 Reagent (11668-019, Invitrogen), according to the manufacturer's instructions. Plasmids were mixed with the transfection reagent and then added collectively to the corresponding cell lines. After 6 h of transfection, the supernatant was replaced with fetal bovine serum (FBS)-containing medium and cells were cultured for 24 h. The establishment of all cell lines was verified using RT-qPCR and western blotting.

### IP and Co-IP

IP and co-immunoprecipitation (Co-IP) were performed using the Pierce Crosslink Immunoprecipitation Kit (26147; Thermo Fisher Scientific) and Pierce Co-Immunoprecipitation Kit (26149; Thermo Fisher Scientific) respectively. The antibodies were coupled with Pierce Protein A/G Plus Agarose or AminoLink Plus Coupling Resin for IP and Co-IP. The cells were then lysed using IP lysis buffer (0.025 M Tris, 0.15 M NaCl, 0.001 M EDTA, and 1% NP-40, 5% glycerol) for 5 min with periodic mixing at 4°C. After centrifugation at 13000 × g for 15 min, the supernatant was immunoprecipitated overnight at 4°C. The precipitated complex was washed with wash buffer and eluted with elution buffer (containing primary amine). The eluotropic samples were analyzed by western blotting.

### PLA

Proximity ligation assay (PLA) was performed using the Duolink® *In situ* Red Starter Kit Mouse/Rabbit (DUO92101; Millipore Sigma, China), according to the manufacturer's instructions. Anti-SMURF2 (sourced from rabbit) and Anti-ID2 (sourced from mouse) antibodies were used to detect specific proteins, which were then stained with DAPI. The PLA signals were visualized using a fluorescence microscope (Nikon, Tokyo, Japan).

### Immunofluorescence staining

Plasmids were transfected, and cells were seeded on coverslips, cultured overnight, and fixed with 4% paraformaldehyde (AR1069, Boster, Wuhan, Hubei, China) for 10 min at room temperature. The cells were permeabilized with 0.2% Triton X-100 (A600198-0500, BBI Life Sciences, Shanghai, China) for 12 min. The cells were blocked with anti-goat blocking medium and then incubated with primary antibodies at 4°C overnight. The slides were washed thrice with phosphate buffered saline (PBS, pH 7.4) and incubated with fluorophore-conjugated secondary antibodies for 1 h at room temperature. Slides were washed thrice with PBS and visualized using a confocal microscope (Nikon).

### Western Blot

All protein extraction steps were performed at 4°C unless otherwise indicated. Radioimmunoprecipitation assay (RIPA) lysis buffer containing phenylmethylsulfonyl fluoride (PMSF) was used to lyse cells. Samples were centrifuged at 13000 × g for 20 min, the supernatants were transferred into new tubes. The Pierce™ BCA Protein Assay Kit (23227; Thermo Fisher Scientific) was used to quantify the protein concentration. The protein samples were separated on 12% SDS-PAGE gels and electrophoretically transferred onto 0.45 µm polyvinylidene fluoride (PVDF, IPVH00010; Millipore) membranes. Then, 5% Bovine serum albumin (BSA, 218054980; MPBio, California, USA) was used to block the membrane for 120 min at room temperature. Subsequently, the membranes were incubated with the primary antibodies at 4°C overnight. The next day, the membranes were equilibrated to room temperature and washed with 1× TBST thrice for 10 min. Secondary antibodies were selected according to the corresponding primary antibodies and incubated for 1 h at room temperature. The membranes with washed with 1× TBST thrice and visualized using Immobilon Western Chemiluminescent HRP Substrate (WBKLS0500, Millipore). Images were captured using a ChemiDoc™ Touch Imaging System (Bio-Rad, California, USA).

### Real-Time Quantitative PCR (qPCR)

A total RNA extraction kit (R6834-1; Omega, China) was used to obtain RNA from cell samples, according to the manufacturer's instructions. Cells were lysed with TRIzol reagent and purified using chloroform. Samples were centrifuged at 13000 g for 15 min, RNA was collected using HiBind™ RNA Columns and quantified using NanoDrop™ 2000c spectrophotometers (Thermo Fisher Scientific). The RNA was then reverse-transcribed to form cDNA using the PrimeScript™ RT Master Mix Kit (RR036A; Takara, China). RT-qPCR was performed using SYBR Premix Ex Taq II (Takara, DRR081A) and the QuantStudio 7 Real-Time PCR System (Thermo Fisher Scientific) with the following primers:

β-actin-Forward: CATGTACGTTGCTATCCAGGC,

β-actin-Reverse: CTCCTTAATGTCACGCACGAT;

SMURF2-Forward: TCCTCGGCTGTCTGCTAACTTG,

SMURF2-Reverse: CAGGCATTCTGTGTCATCAGGAC;

ID2-Forward: TTGTCAGCCTGCATCACCAGAG,

ID2-Reverse: AGCCACACAGTGCTTTGCTGTC;

CDKN1A-Forward: AGGTGGACCTGGAGACTCTCAG,

CDKN1A-Reverse: TCCTCTTGGAGAAGATCAGCCG;

CDK2-Forward: ATGGATGCCTCTGCTCTCACTG,

CDK2-Reverse: CCCGATGAGAATGGCAGAAAGC;

CDK4-Forward: CCATCAGCACAGTTCGTGAGGT,

CDK4-Reverse: TCAGTTCGGGATGTGGCACAGA;

CDK6-Forward: GGATAAAGTTCCAGAGCCTGGAG,

CDK6-Reverse: GCGATGCACTACTCGGTGTGAA.

### Dual luciferase reporter assay

A549 and H460 cells were seeded in 24-well plates and co-transfected with CDKN1A luciferase reporter, Renilla luciferase reporter, and E2A with or without the ID2 plasmid. Luciferase activity was measured using the Dual-Luciferase^®^ Reporter Assay System Kit (E1980; Promega, USA) 24 h after transfection, according to the manufacturer's instructions. All data are presented as the mean ± standard deviation (SD) of at least three experiments.

### ChIP

A commercial EZ-Magna ChIP A/G Chromatin Immunoprecipitation kit (17-10086, Millipore) was used to perform the ChIP assay, according to the manufacturer's instructions. A549 cells (1 × 10^7^ cells) were transfected with the Flag-E2A plasmid for 24 h and then cross-linked in 1% formaldehyde. The reaction was aborted using glycine buffer and cells were lysed by sonication (Bioruptor UCD-200) for 5 min (30-s sonication, 10 cycles) at 4°C. The DNA fragments obtained were confirmed by agarose gel electrophoresis and then incubated with beads and anti-Flag antibodies at 4°C overnight to obtain DNA-protein complexes. Samples were treated with RNase A and proteinase K, and the immunoprecipitated DNA fragments were identified by qPCR using the primer sequences from Tsingke Biotechnology Co., Ltd. (Beijing, China) for the E2A protein combination. The primers used were as follows: ChIP forward (5'-3'), GACAGGTTGTAGATTGCCAGC; and ChIP reverse (5'-3'), TTCAGGAATGCCGCAGATGT.

### Cell cycle analysis

Cell cycle analysis was performed using a Cell Cycle Detection Kit (KGA512, Nanjing Keygen Biotech Co., Ltd., Jiangsu, China) and flow cytometric evaluation. Cells were harvested and fixed in 4 mL of ice-cold 70% ethanol for 6 h. The cells were routinely washed with PBS and stained with 200 μL staining solution consisting of propidium iodide (PI) and RNase A at room temperature in the dark. The samples were subsequently analyzed using a FACSCalibur™ Flow Cytometer (BD Biosciences, San Jose, CA, USA). The experimental data were analyzed by professional technicians of our laboratory.

### CCK-8 assay

Trypan blue (T10282, Thermo Fisher Scientific) was used to assess cell count and viability. Cell proliferation was assessed using a cell counting (CCK-8) kit (C0005, Topscience, Rizhao, Shandong, China). SMURF2-KD cells (3 × 10^3^ cells/well) were seeded in 96-well plates, and the absorbance (450 nm) was measured at the indicated time points (0, 24, 48 and 72 h) before incubation with CCK-8 solution for 2 h at 37°C.

### Cell migration and invasion assays

The migration and invasion ability of the cells were detected using 24-well Millicell chambers (PIHP01250, Millipore) and for the invasion assay the chambers were precoated with Matrigel (354234, Corning). A total of 1 × 10^4^ serum-starved cells were added to the upper side of the chamber and then placed in medium containing 10% serum in a 24-well plate. Cells were incubated at 37°C for 24 h, and the cells on the lower membrane surface were stained with 0.1% crystal violet for visualization. Invasion and migration of cells were quantified in three randomly selected fields.

### Wound healing assay

The cells were seeded in 24-well plates and grown to approximately 90% confluence. A sterile pipette tip was used to create the scratch wounds, which were then cultured in serum-free media for 48 h at 37°C with 5% CO_2_. The wounded areas were imaged at the set time point, and ImageJ Fiji (WS Rasband, National Institute of Health, Bethesda, MD) was used to calculate the degree of wound repair.

### Establishment of NSCLC nude mice model

The animal experiments in this study were approved by the National Translational Science Center for Molecular Medicine of Institutional Animal Care and Use Committee (IACUC) of the Fourth Military Medical University. Five-week-old BALB/c nude male mice were obtained from Vital River Laboratory Animal Technology Co., Ltd. (Beijing, China). Mice were acclimated for one week, subcutaneously injected with 1 × 10^7^ A549-shSMURF2 and A549-shNC cells in 100 μL RPMI 1640 medium, and the tumor volume was measured every two days for two weeks. Tumor volume (mm^3^) = 0.5 × (tumor width)^2^ × (tumor length). The tumor weight was measured at the end of the experiment.

### Immunohistochemical staining

For immunohistochemical (IHC) staining, formaldehyde-fixed, paraffin-embedded sections of tumor tissues were obtained from mouse models. Briefly, the deparaffinized sections were then treated with methanol containing 3% hydrogen peroxide for 15 min. The sections were washed with PBS and blocked with blocking serum for 30 min at room temperature. The sections were then incubated with primary antibodies, including anti-SMURF2 (bs-4056R, Bioss), anti-ID2 (MA5-32891, Invitrogen), and anti-p21 (ab107099, Abcam) at 4°C overnight. The following day, the primary antibodies were washed off and sections were incubated with HRP-conjugated secondary antibodies for 2 h at room temperature. The nuclei were stained with hematoxylin, and DAB substrate (ZLI-9019, ZSGB-Bio, Beijing, China) was added to detect the proteins. The obtained immunohistochemistry images were analyzed with following scoring criteria. Cells with 0% staining were scored as 0; cells with 1%-33% staining were scored as 1; cells with 34%-66% staining were scored as 2; cells with 67%-100% staining were scored as 3. Additionally, the staining intensities were evaluated into four grades: 3 (strong), 2 (moderate), 1 (weak) and 0 (none). The final score was defined as the score of percentage classifications multiplied by intensity grades.

### Bioinformatics analysis

SMURF2 expression and its prognostic value in lung adenocarcinoma (LUAD) were evaluated using the cancer genome atlas (TCGA) database. Three additional GSE datasets (GSE75037, GSE149507, and GSE130779) were used to assess the relative expression of SMURF2 in tumors and non-tumors. The three-dimensional structure of SMURF2 (PDB ID: 7M3Q) was downloaded from the Protein Data Bank (http://rcsb.org/). The online tool ZDOCK (https://zdock.umassmed.edu/) was used to predict protein-protein docking, and PyMol software (version 2.5.2, https://pymol.org/2/) was used for visualization. The biological general repository for interaction datasets (BioGRID, https://thebiogrid.org/) is a public database that archives and disseminates genetic and protein interaction data from model organisms and humans. ID2-interaction proteins were identified by the BioGRID dataset in this study as well as GeneCards (www.genecards.org) database.

### Statistical analysis

All quantitative data were repeated at least three times. Differences between two groups were evaluated by performing unpaired Student's *t*-tests using GraphPad Prism version 9.0 (GraphPad Prism Software Inc., San Diego, CA, USA). Comparisons between more than two groups were performed using one-way analysis of variance. Spearman's correlation analysis was used to describe the correlation between quantitative variables without a normal distribution. Data are presented as means ± standard deviation (SD), and statistical significance was set at *P* < 0.05.

## Results

### SMURF2 expression is decreased in LUAD and correlated with favorable prognosis

**Figure 1:**
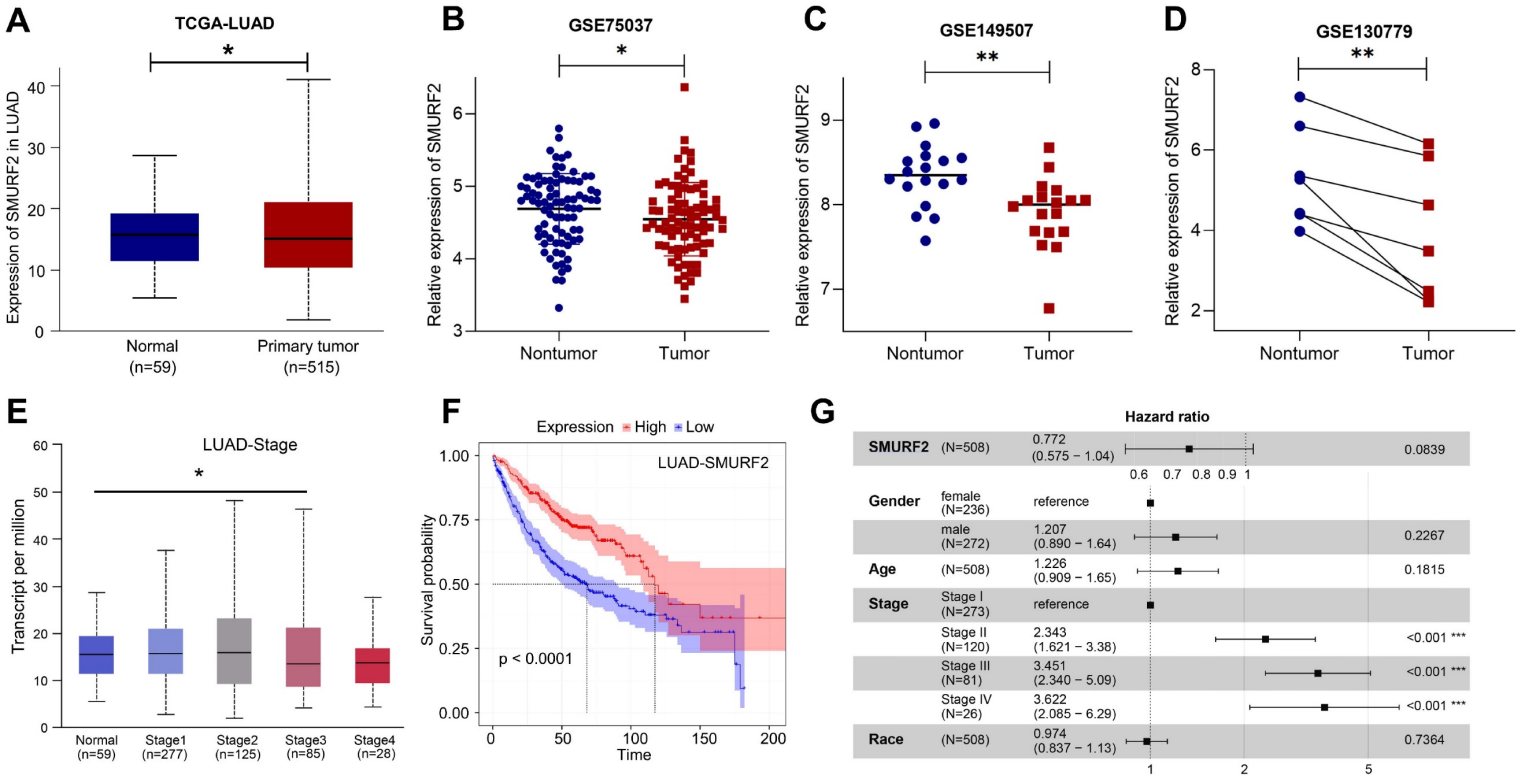
** SMURF2 expression and its diagnostic and prognostic value in patients with lung cancer. (A)** Data from TCGA-LUAD revealed that SMURF2 was downregulated in tumor tissues. **(B-D)** The expression of SMURF2 in lung tumor tissues compared to adjacent normal tissues in GSE75037, GSE149507, GSE130779 (dropped one case of outliers). **(E)** SMURF2 expression based on tumor stage of patients with LUAD. **(F)** Kaplan-Meier curve was performed to analyze the prognosis value of SMURF2 in patients with LUAD. **(G)** SMURF2 expression as well as several key clinical covariates were analyzed by cox proportional hazard model based on TCGA lung adenocarcinoma (n = 508 patients).^ *^P < 0.05; ^**^P < 0.01.

The data from the HPA and TCGA databases revealed that the E3 ubiquitin ligase SMURF2 was highly expressed in normal human lung tissues (Figure S1A). Among the multiple tumor types, LUAD exhibited high SMURF2 expression (Figure S1B). These results suggest a possible association between SMURF2 and the development of LUAD. The expression profiles of SMURF2 in patients with LUAD were evaluated using the TCGA database. Significantly downregulated expression of SMURF2 was observed (Figure 1A), and this result was further validated by data from three GEO datasets (Figure 1B-D). The relationship between SMURF2 expression and tumor grade in LUAD was evaluated by UALCAN database (http://ualcan.path.uab.edu/index.html), and the results suggested that patients with LUAD at stage three exhibited low SMURF2 expression (Figure 1E, *P* < 0.05). However, there was no statistical difference in SMURF2 expression between patients with LUAD at stage four and normal tissues (Figure 1E, *P* = 0.374), which may be due to the limited number of patients in the database. Moreover, the effect of SMURF2 expression on the survival rate of patients with LUAD was evaluated and high SMURF2 expression was observed to be significantly associated with favorable overall survival (Figure 1F). The diagnostic value of SMURF2 and several key clinical covariates were calculated in LUAD patients, and the forest plot (Figure 1G) exhibited the detailed hazard ratio of SMURF2 (HR = 0.772; confidence interval, 0.575-1.04; *P* = 0.084). Although the *P*-value did not satisfy the significance criteria (*P* < 0.05) in this study, this result suggests a protective role of SMURF2 in LUAD tumorigenesis. More studies with larger patient sample sizes or stricter grouping criteria may provide more information. Taken together, these results suggest that SMURF2 may function as a tumor suppressor in lung adenocarcinoma.

### SMURF2 regulates the expression profiles of proliferation-related genes

**Figure 2:**
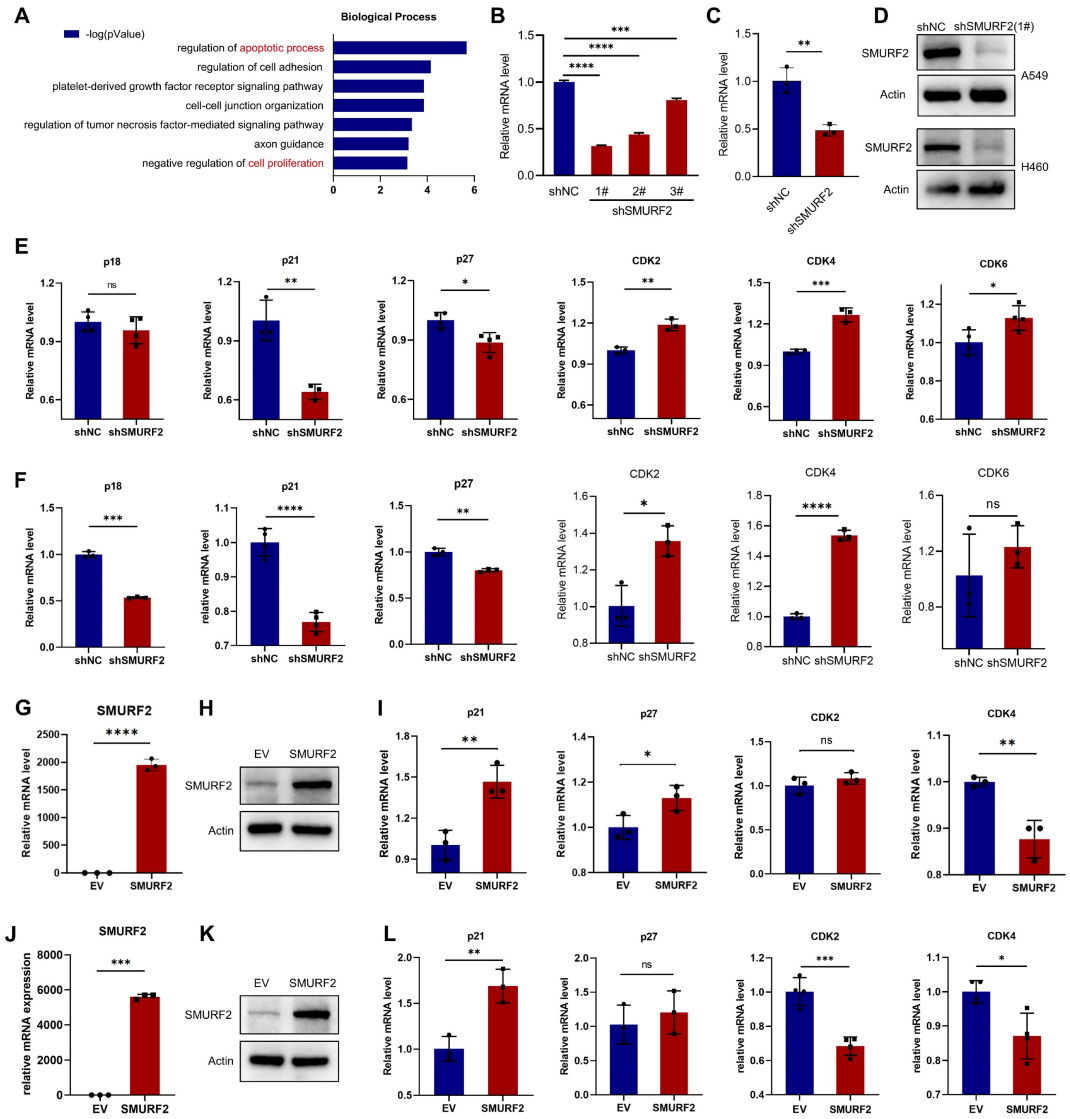
**The effects of SMURF2 on the expression of cell cycle proteins in lung cancer cell lines. (A)** Biological processes enrichment analysis of the target genes co-expressed with SMURF2. **(B-D)** RT-qPCR and WB analysis of SMURF2 expression in SMURF2-KD A549 (b) and H460 (c) cells. **(E, F)** Detection of mRNA level of p18, p21, p27, CDK2, CDK4 and CDK6 in SMURF2-KD A549 (e) and H460 (f) cells. **(G, H)** SMURF2-OE efficiency of A549 cell was detected by RT-qPCR (g) and WB (h). **(I)** Detection of mRNA level of p21, p27, CDK2 and CDK4 in SMURF2-OE A549 cells. **(J, K)** SMURF2-OE efficiency of H460 cell was detected by RT-qPCR (j) and WB (k). **(L)** Detection of mRNA level of p21, p27, CDK2 and CDK4 in SMURF2-OE H460 cells.^ *^P < 0.05; ^**^P < 0.01; ^***^P < 0.001; ^****^P < 0.0001; ns, no significance.

The potential tumor suppression mechanisms of SMURF2 in LUAD were investigated. The online Coexpedia tool (https://www.coexpedia.org/) 23 was used to investigate the genes co-expressed with SMURF2 and assess their biological roles in tumors. Enrichment analysis of target genes was performed (Figure 2A). A negative regulation of proliferation was identified in the analysis. Accordingly, we speculated that the negative control of cell growth by SMURF2 may explain its tumor-suppressive effect on LUAD. This hypothesis was tested by investigating the effects of SMURF2 on several cell cycle-related genes using KD or OE lung cancer cell lines (A549 and H460). Infection efficiency was evaluated using green fluorescent proteins in A549 (Figure S2A) and H460 cells (Figure S2B) cells and then three shRNAs against SMURF2 in A549 and H460 cells were confirmed by WB (Figure S2C). SMURF2 mRNA and protein levels were knocked down in A549 (Figure 2B, D) and H460 (Figure 2C-D) cells and validated by qPCR and western blotting, respectively. Several genes related to cell cycle progression were evaluated, including CDKN2C (p18), CDKN1A (p21), CDKN1B (p27), CDK2, CDK4, and CDK6. The expression of Cdk inhibitors (CKIs, p21, and p27) was significantly decreased following SMURF2 KD, while Cdks were increased in both A549 (Figure 2E) and H460 (Figure 2F) cells. SMURF2-encoding plasmids were transfected into A549 (Figure 2G-H) and H460 (Figure J-K) cells and their overexpression validated by qPCR and western blotting. Significantly increased mRNA levels of CDKN1A and decreased levels of CDK4 were observed in both A549 (Figure 2I) and H460 (Figure 2L) cells. Cell cycle is a tightly regulated series of events controlled by multiple proteins, of which CDK4 specifically regulates the cell cycle transition from G1 to S phase 24. p21 associates with Cdk/cyclin complexes and inhibits their kinase activities at the G1/S and G2/M phases 25. These results suggest that the tumor-suppressive effect of SMURF2 may result from cell cycle arrest by influencing the expression of Cdks and CKIs.

### SMURF2 depletion promotes cell proliferation and tumorigenesis* in vitro*

**Figure 3:**
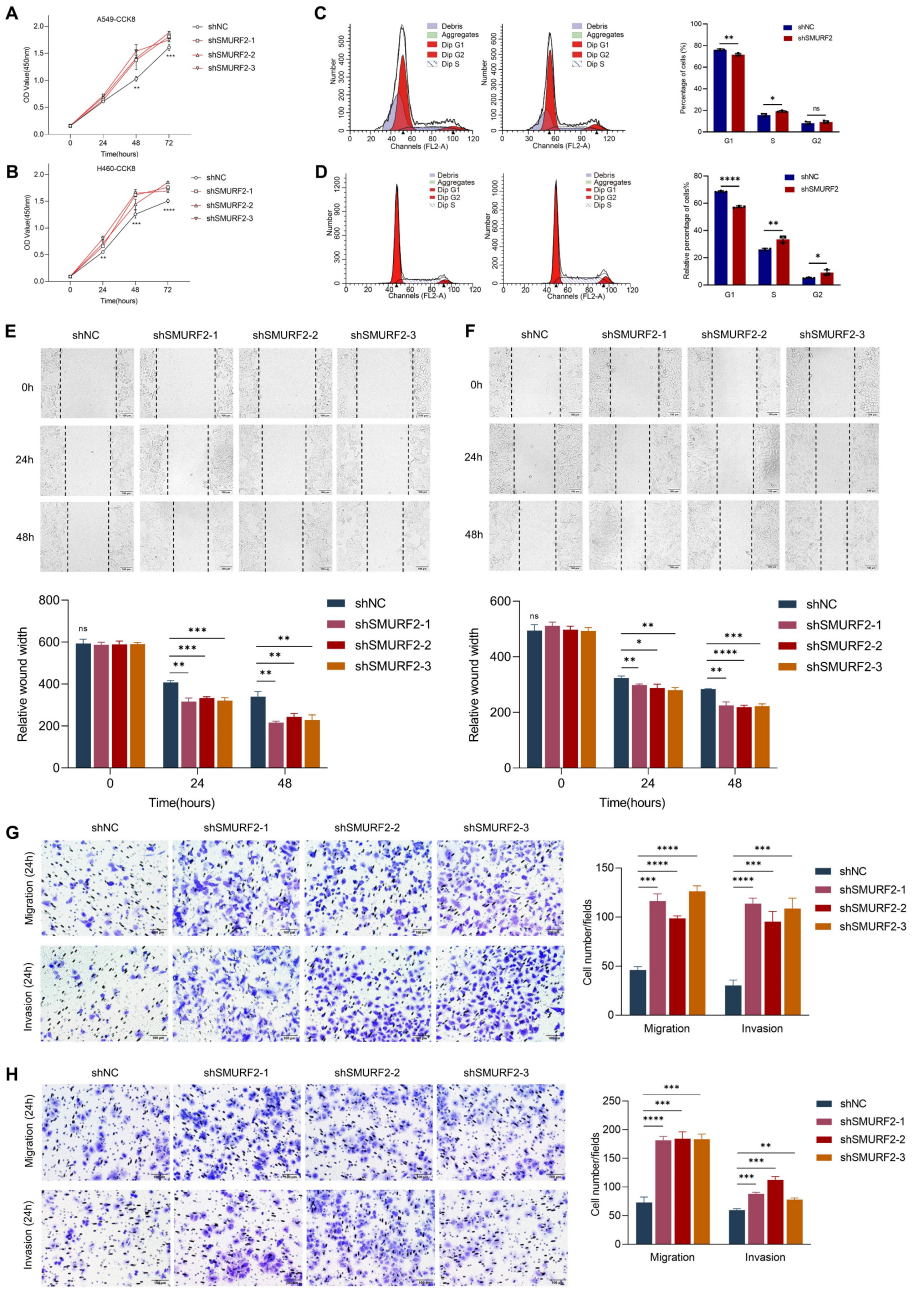
** SMURF2 knockdown promotes cell proliferation and tumorigenesis. (A, B)** Cell proliferation was monitored by CCK-8 assay of A549 and H460 cells at the indicated time points (0 h, 24 h, 48 h and 72 h). **(C)** A549 cell cycle was determined by flow cytometry with quantitative analysis (right panel). **(D)** H460 cell cycle was determined by flow cytometry with quantitative analysis (right panel). **(E)** Cell migration of A549 cells were assessed by wound healing assay with quantitative analysis (bottom panel). **(F)** Cell migration of H460 cells were assessed by wound healing assay with quantitative analysis (bottom panel). **(G)** Migration and invasion of A549 cells were monitored by transwell assay with quantitative analysis (right panel). **(H)** Migration and invasion of H460 cells were monitored by transwell assay with quantitative analysis (right panel).^ *^P < 0.05; ^**^P < 0.01; ^***^P < 0.001;^ ****^P < 0.0001; ns, no significance; scale bar, 100 μm.

Cell proliferation-related genes suggested a possible tumor-suppressing role for SMURF2, whereas the detailed function of this ubiquitin ligase remained unclear. The effects of SMURF2 on the biological behavior of lung cancer cell lines were investigated by performing cell proliferation, migration, and invasion analyses using the constructed cells with SMURF2 KD. CCK-8 assays confirmed that SMURF2 KD drastically promoted the proliferation of A549 (Figure 3A) and H460 (Figure 3B) cells. Flow cytometry was used to further elucidate the effects of SMURF2 on cell cycle progression. Depletion of SMURF2 in A549 cells significantly induced the transition from G1 to S phase (Figure 3C), and the same results were observed in H460 cells (Figure 3D). Overexpression of SMURF2 in A549 (Figure S3A) and H460 (Figure S3B) cells resulted in cell cycle arrest in the G1/S phase. Additionally, cell migration ability was assessed using wound healing assay. In both A549 (Figure 3E) and H460 (Figure 3F) cells, the SMURF2-KD groups exhibited a dramatically increased ability to migrate. We further performed transwell assay with SMURF2 KD in A549 (Figure 3G) and H460 (Figure 3H) cells and revealed that cell invasion was also accelerated by SMURF2 KD.

More importantly, ID2 expression was reduced by three siRNAs in A549 and H460 cells and then confirmed by WB (Figure S3C). Downregulation of ID2 in SMURF2-knockdown cells resulted in the decreased capacity of migration (Figure S3D) and proliferation (Figure S3E), and overexpression of E2A restored p21 expression following SMURF2 knockdown (Figure S3F). In conclusion, these data suggest that SMURF2 downregulation promotes lung cancer cell proliferation, accelerates their migration and invasion, and may ultimately contribute to tumor progression.

### SMURF2 interacts with ID2

**Figure 4:**
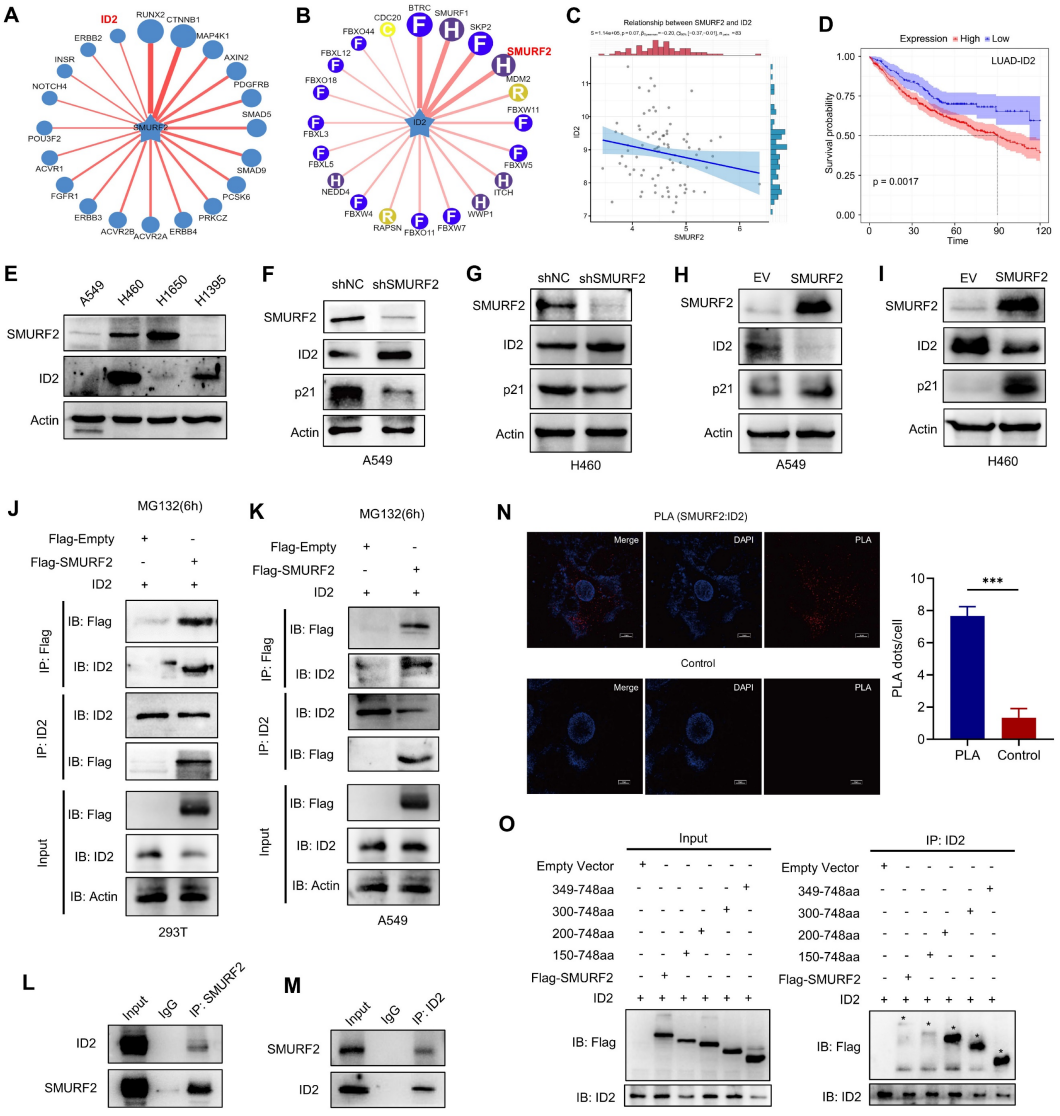
** SMURF2 interacts with ID2. (A)** Substrate protein prediction for SMURF2 using an online tool. **(B)** Prediction of the E3 ubiquitin ligase for ubiquitinating ID2. **(C)** The expression correlations between SMURF2 and ID2 was analyzed. The value on the top represents the correlation *p* value, correlation coefficient and correlation calculation method. **(D)** Kaplan-Meier curve revealed the correlation between ID2 expression and the outcomes of patients with LUAD. **(E)** Relative expression of SMURF2 and ID2 in A549, H460, H1650 and H1395 cells. **(F, G)** WB analysis of the protein expression following SMURF2-KD in A549 and H460 cells. **(H, I)** WB analysis of the protein expression following SMURF2-OE in A549 and H460 cells. **(J, K)** The interaction between SMURF2 and ID2 was identified by immunoprecipitating with anti-Flag antibody and anti-ID2 antibody in HEK293T and A549 cells. **(L, M)** Endogenous interaction was detected by IP with anti-SMUFR2 and anti-ID2 antibodies in A549 cells. **(N)** PLA assay detected the interaction between ID2 and SMURF2. A red fluorescent signal indicates a distance between SMURF2 and ID2 smaller than 40 nm and the quantification of PLA dots per cell was analyzed. Scale bar, 10 μm. **(O)** Truncated mutants of human SMURF2 revealed that ID2 binds to the HECT domain of SMURF2. ^***^P < 0.001.

SMURF2 generally functions through ubiquitin-mediated degradation of substrate proteins. Therefore, the UbiBrowser (http://ubibrowser.bio-it.cn/ubibrowser/) 26 tool was used to predict the possible substrates of SMURF2, especially the proteins related to cell cycle regulation (Figure 4A). Notably, ID2 was predicted to be a substrate for SMURF2 with a confidence score of 0.736, and the obtained image was marginally modified based on the database. ID2 is a member of the four DNA-binding protein inhibitors ID1-4, which have been identified with extensive sequence homology in their HLH motif 27. As an inhibitor of E proteins, ID2 is capable of inhibiting the differentiation of multiple cell types, promoting cell cycle progression, delaying cellular senescence, and facilitating cell migration 28. SMURF2 was predicted to degrade ID2 (Figure 4B), which supports the possibility of SMURF2-mediated ID2 downregulation. The relationship between SMURF2 and ID2 expression was further analyzed using the TCGA database (https://portal.gdc.cancer.gov/). Spearman's correlation analysis revealed a possible negative correlation between these two proteins (*P* = 0.07; Figure 4C). Survival analysis based on TCGA-LUAD patients revealed that high expression of ID2 was typically associated with poorer survival probability (Figure 4D). Results from these databases revealed a possible ubiquitin-mediated negative relationship between SMURF2 and ID2, and ID2 may play a tumor-promoting role in LUAD, as has been previously demonstrated in other cancers 12, 17, 27, 29.

Subsequently, the potential interaction between SMURF2 and ID2 was explored. Four lung cancer cell lines, A549, H460, H1650, and H1395, were used to explore the relationship between SMURF2 and ID2 expression. Differential expression between these two proteins was observed among the four types of cancer cells, which suggested possible negative expression profiles among them (Figure 4E). Additionally, several studies have suggested an increased p21 expression following ID2 loss in tumors 17, 30, 31. ID2 expression was significantly upregulated and p21 expression was significantly decreased after SMURF2 KD in A549 (Figure 4F) and H460 (Figure 4G) cells. These results were also confirmed by SMURF2 overexpression in lung cancer cells (Figure 4H-I). Co-IP assays were performed to explore the interaction between SMURF2 and ID2. HEK293T and A549 cells were co-transfected with Flag-SMURF2 and ID2 plasmids, and IP was performed using an anti-Flag antibody and anti-ID2 antibody. The results showed that SMURF2 interacted with ID2 (Figure 4J-K). We further confirmed endogenous interactions between SMURF2 and ID2 by IP with anti-SMUFR2 (Figure 4L) and anti-ID2 antibodies (Figure 4M) in A549 cells.

Proximity ligation assay was performed to further validate this interaction A significant PLA signal was observed in A549 cells after PLA probe ligation (Figure 4N). Furthermore, colocalization of SMURF2 and ID2 was detected in A549 and H460 cells (Figure S4A). SMURF2 contains one N-terminal C2 domain, three tryptophan-tryptophan (WW) domains, and a HECT domain (Figure S4B) 32. However, the ID2 protein features a relatively less sophisticated structure, which lacks the basic DNA binding domain but contains a HLH motif (Figure S4B) 28, 33.

The detailed interaction between SMURF2 and ID2 was shown in Figure S4C. We generated truncated fragments of SMURF2 and co-transfected these with GFP-ID2 into HEK 293T cells. Cell lysate IP with anti-Flag antibodies showed that the HECT domain of SMURF2 probably played an essential role in the interaction with ID2 (Figure 4O). Truncated fragments of SMURF2 and GFP-ID2 were co-transfected into HEK293T cells and immunoprecipitated with an anti-ID2 antibody. The same conclusion was reached with the anti-Flag antibody (Figure S4D). Taken together, these data suggest that SMURF2 directly interacts with ID2.

### SMURF2 regulates the ubiquitination of ID2

**Figure 5:**
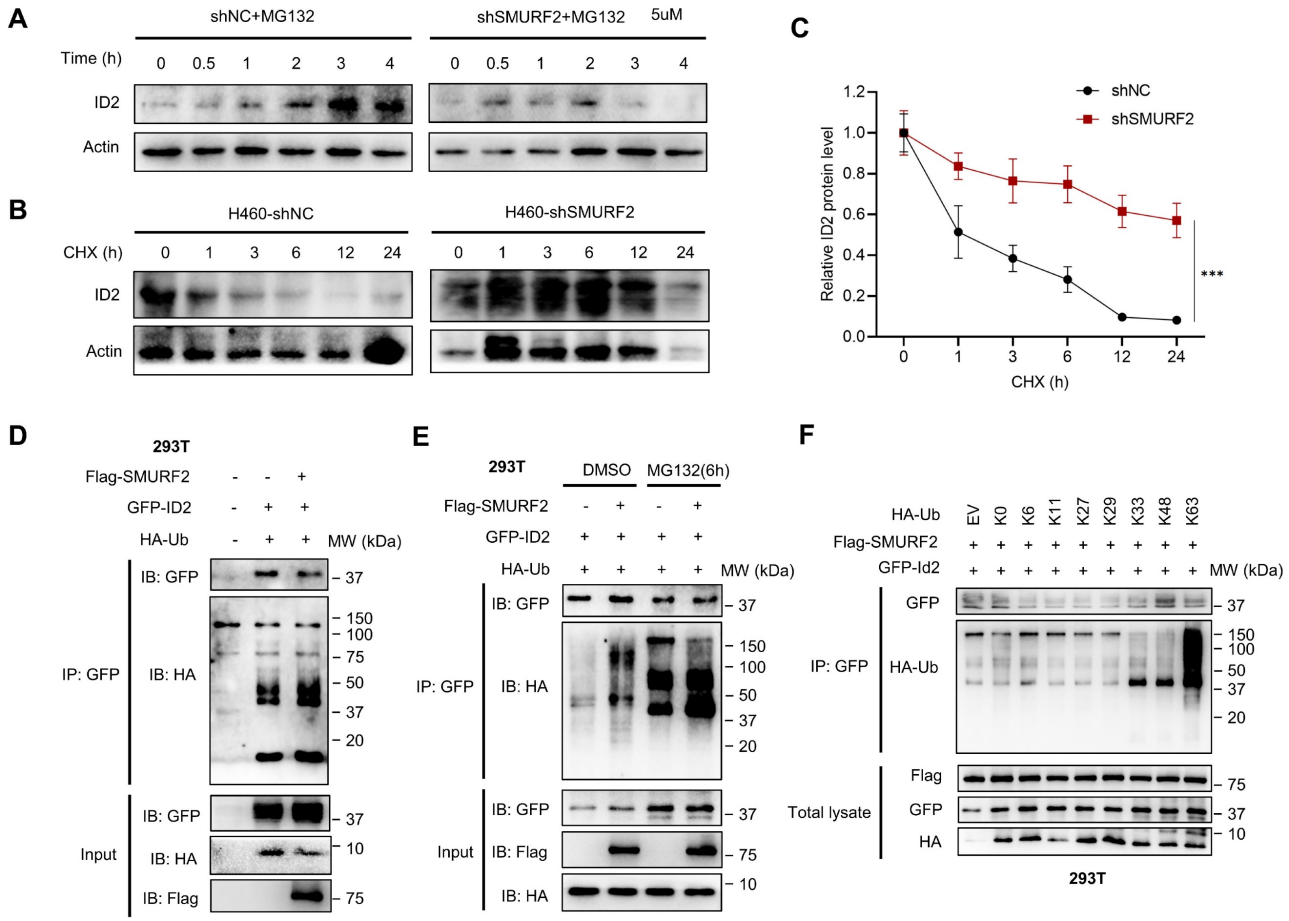
** SMURF2 regulates the ubiquitination of ID2. (A)** The expression level of ID2 was detected following SMURF2-KD in A549 cells. **(B, C)** Detection ID2 protein level in the presence of CHX following SMURF2-KD in H460 cells, and the quantitative result was presented. **(D, E)** The ubiquitination level of ID2 was detected in HEK293T cells and further detected with the use of MG132. **(F)** WB analysis of the ubiquitination types of ID2.^ ***^P < 0.001.

A previous study showed that ID2 is normally poly-ubiquitinated and can be rapidly degraded by the ubiquitin proteasome pathway (UPS) 12. To further demonstrate the possible regulatory mechanisms of SMURF2 on ID2, we used MG132 and CHX to inhibit degradation and block synthesis in lung cancer cells. Treatment with MG132 antagonized SMURF2-mediated degradation, which resulted in the increased speed and proportion of ID2 in the control group being greater than in the SMURF2-KD group (Figure 5A). In view of the protein level of ID2 decreased dramatically after 3 hours of MG132 administration, additional protein degradation mechanism should be involved in the degradation of ID2, such as autophagy, which needs to be tested with additional research.

Additionally, CHX was used to block protein translation in both groups, and western blotting revealed that the half-life of ID2 was significantly increased in SMURF2-KD cells (Figure 5B). The quantification results showed a significant loss of proteasome-dependent degradation of ID2 after SMURF2 deletion (Figure 5C). Furthermore, the mRNA levels of ID2 exhibited no significant changes in A549 (Figure S5A) and H460 (Figure S5B) cells, further validating the role of SMURF2 in mediating ID2 protein degradation. Notably, co-expression of Flag-SMURF2, GFP-ID2, and HA-ubiquitin in HEK 293T cells resulted in a remarkably increased level of ubiquitinated ID2 (Figure 5D). MG132 further consolidated the changes in the expression of these proteins, and ID2 ubiquitination was significantly increased (Figure 5E). Immunoprecipitation with ID2 using SMURF2 knockdown A549 cells revealed a significant decrease in the ubiquitination of ID2 (Figure S5C). Notably, a single-point tyrosine retention mutation (with the remaining lysine residues mutated to arginine) assay showed K33, K48, and K63 linked ubiquitination were observed in ID2 (Figure 5F). To summarize, these data suggest that SMURF2 mediates ID2 ubiquitination and degradation.

### ID2 inhibits E2A-mediated transcriptional activation of p21 expression

**Figure 6:**
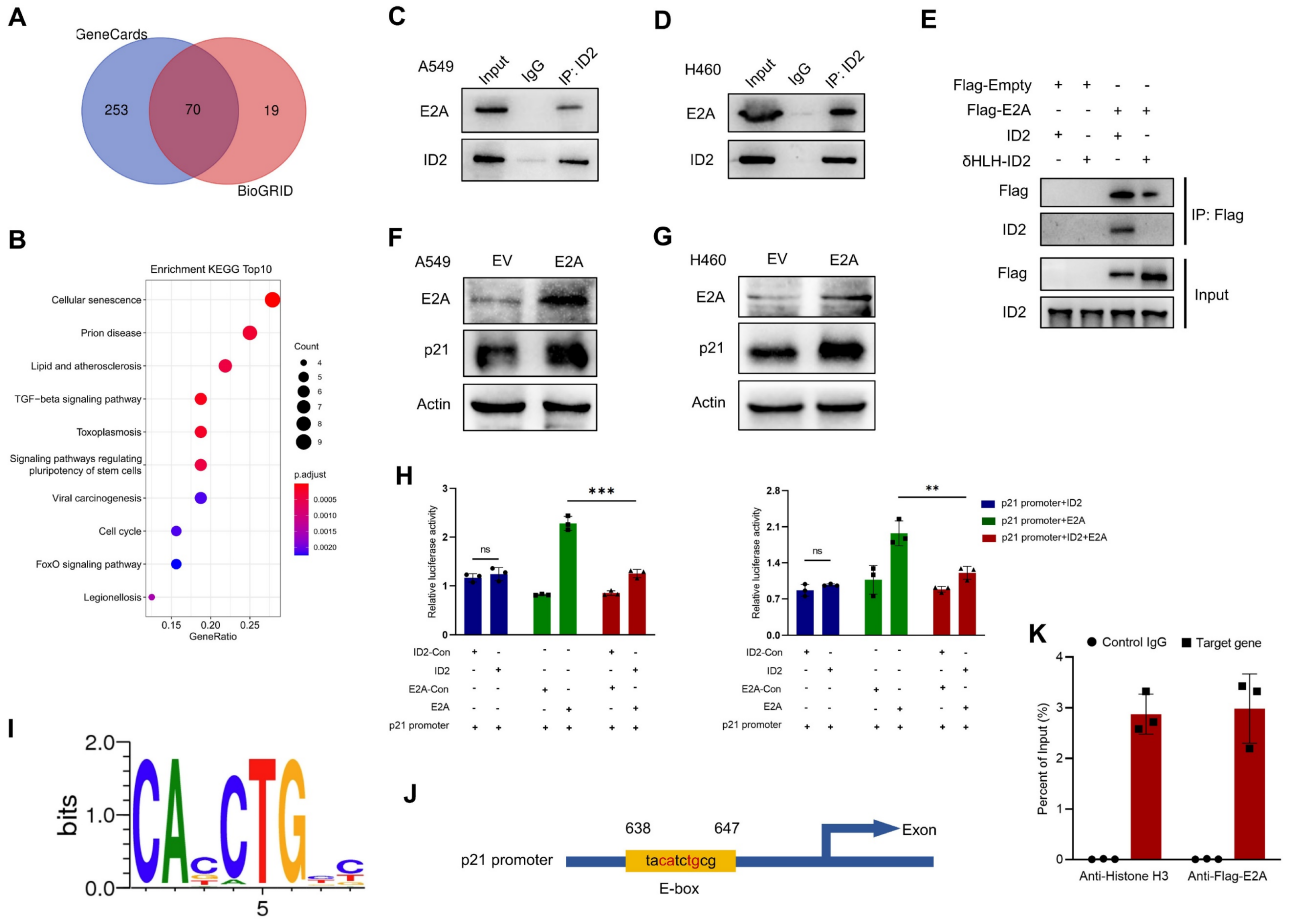
** ID2 inhibits E2A-mediated transcriptional activation of p21 expression. (A)** Venn diagram was drawn to identify the possible interacted genes of ID2. **(B)** The Common genes derived from Venn were used to perform KEGG enrichment analysis. **(C, D)** Endogenous interaction between ID2 and E2A was detected in A549 and H460 cells. **(E)** The interaction domain between ID2 and E2A was identified by WB analysis. **(F, G)** p21 expression was detected following E2A OE in A549 and H460 cells. **(H)** Relative luciferase activity of CDKN1A promoter region in A549 (left) and H460 (right) cells. **(I)** Evolutionarily conserved E-box binding sites. **(J)** The predicted E2A binding site in the CDKN1A locus. **(K)** ChIP assay was used to illustrate the transcriptional regulation of CDKN1A.^ **^P < 0.01; ^***^P < 0.001.

And then the possible regulatory mechanisms of ID2 in tumors were investigated. Bioinformatics analyses of BioGRID database identified 135 genes which interact with ID2. Meanwhile, 323 interaction-related genes were identified from GeneCards. An online tool (http://bioinformatics.psb.ugent.be/webtools/Venn/) was used to analyze and draw the Venn diagram (Figure 6A). The intersection of these genes was used to perform a Kyoto encyclopedia of genes and genomes (KEGG) enrichment analysis (Figure 6B). Consistent with previous observations, ID2 was involved in cell cycle regulation, acting through its transcription-factor activity. ID2 is a negative regulator of the E protein TF family 34. Its abundance plays a decisive role in determining whether E proteins can bind to the E-box (CANNTG) and participate in transcriptional regulation 35, 36. E2A, a member of the E protein TF family, directly regulates the cell cycle by upregulating CDKN1A (p21) transcription. We attempted to determine the relationship between ID2, E2A, and p21. First, an IP assay showed an endogenous interaction between ID2 and E2A in A549 (Figure 6C) and H460 (Figure 6D) cells. The HLH domain of ID2 was responsible for this interaction (Figure 6E). Second, overexpression of E2A in A549 and H460 cells confirmed that E2A can positively regulate the expression of p21 (Figure 6F-G). Moreover, the elevated mRNA level of p21 was detected in A549 and H460 cells following the overexpression of E2A (Figure S6A). Subsequently, dual luciferase reporter assays confirmed that E2A significantly enhanced CDKN1A transcription in both A549 and H460 (Figure 6H) cells. The co-transfection of E2A and ID2 in these two cell lines significantly reduced the transcriptional activity of E2A on CDKN1A. Increased ID2 protein levels in lung cancer cells did not further alter CDKN1A transcriptional activity (Figure S6B). Finally, we searched for evolutionarily conserved E-box binding sites in the CDKN1A locus (Figure 6I) and identified a single E2A consensus binding site in the promoter region of CDKN1A (Figure 6J). ChIP assay was performed to explore the relationship between E2A and CDKN1A. Ultrasonic DNA fragmentation was detected using agarose gel electrophoresis (Figure S6C). The ChIP assay demonstrated that E2A regulates CDKN1A transcription (Figure 6K). These data suggest that E2A positively regulates p21, and that this regulation is blocked by ID2 expression. ID2 inhibits p21 expression by blocking E2A binding to the E-box in the CDKN1A promoter.

### SMURF2 depletion promotes tumorigenesis in LUAD* in vivo*

**Figure 7:**
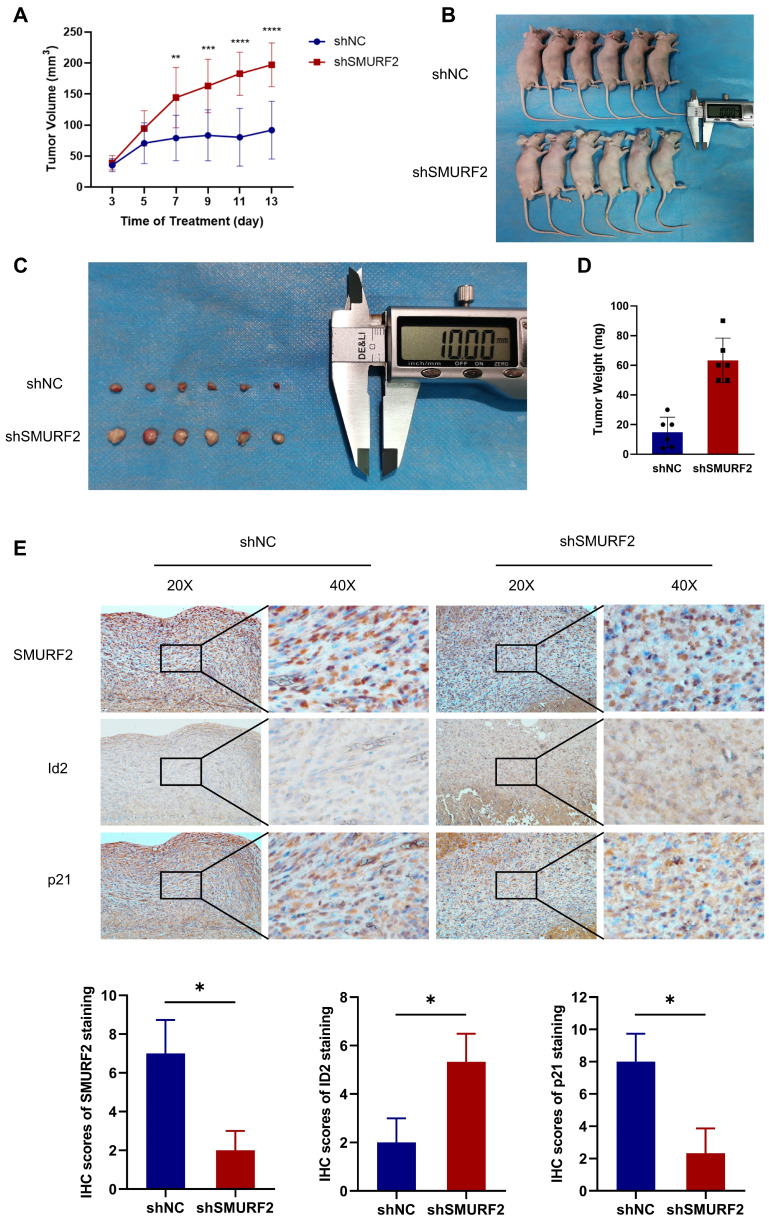
** SMURF2 depletion promotes the formation and development of xenograft tumors. (A)** Tumor volumes were measured and calculated every two days between SMURF2-NC and SMURF2-KD groups. **(B)** Images of tumor-bearing mice were captured from SMURF2-NC and SMURF2-KD groups. **(C)** Representative images of tumors obtained from SMURF2-NC and SMURF2-KD groups. **(D)** Tumor weight was assessed at the end time points the experiments. **(E)** Typical immunohistochemical images of tumor tissues exhibited the expression of SMURF2, ID2 and p21.^ *^P < 0.05; ^**^P < 0.01; ^***^P < 0.001; ^****^P < 0.0001.

**Figure 8:**
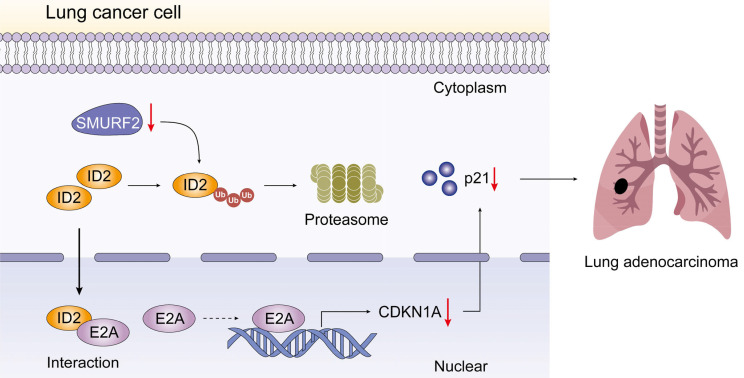
Schematic depiction of the regulatory mechanisms of SMURF2 in lung cancer cell.

The effects of SMURF2 on tumorigenesis were evaluated *in vivo* using tumor-bearing assays in nude mice. Depletion of SMURF2 resulted in a significant increase in xenograft tumor development and volume (Figure 7A-B). The mice were anesthetized and sacrificed, and tumor tissues were isolated, photographed (Figure 7C), and weighed (Figure 7D). IHC staining demonstrated that the expression of ID2 was relatively high and that of p21 was low after SMURF2 KD (Figure 7E). In conclusion, our findings suggest that SMURF2 prevents tumor progression by regulating the cell cycle in NSCLC cells (Figure 8).

## Discussion

Understanding and characterizing PTM, an important approach of increasing proteome diversity and maintaining cellular homeostasis, has greatly increased our understanding of cancer biology 37. In this study, we propose that E3 ubiquitin ligase, SMURF2, is downregulated in NSCLC cells. SMURF2 directly interacts with ID2 and mediates its ubiquitination via the ubiquitin-proteasome pathway. The upregulated ID2 leads to a decrease in E2A, causing transcriptional downregulation of p21, and finally promoting the progression of lung tumors.

Ubiquitination is a reversible modulation and PTM of proteins that regulates protein degradation through the proteasome, altering their localization, affecting their activity, and promoting or interfering with protein interactions 38. The fundamental steps of this modification require the participation of a small evolutionarily conserved protein, the 76-amino acid protein ubiquitin (Ub), to link to the substrate protein 39. Ubiquitin chains can be conjugated to target proteins at specific amino acid residues through different initiatives, including mono-ubiquitination by a single ubiquitin molecule and poly-ubiquitination by linking individual ubiquitin molecules to internal lysine residues (K6, K11, K27, K29, K33, K48, and K63) or amino-terminal methionine 39, 40. These eight types of linkages co-exist in cells and have been shown to play distinct roles in almost all aspects of eukaryotic biology.

K48-linked ubiquitination mediated by SMURF2 has been characterized and found to play a role in a variety of diseases, including tumors 41, 42. Typically, Lys48-linked ubiquitin chains are the best-characterized type of linkage and have long been recognized to drive the proteasomal degradation of certain proteins 43. In the present study, we found that SMURF2 mediated K33, K48, and K63 linked ubiquitination of ID2, and thus caused proteasomal pathway degradation and rapid regulation of ID2 protein levels in NSCLC cells. Notably, other atypical ubiquitin types, such as Lys6, Lys11, Lys27, Lys33, Lys29, and Lys63, also play various non-degradation roles in the regulation of multiple intracellular signals 44-47, such as mitochondrial homeostasis 48-50, cell cycle regulation 51, 52, T-cell activation 53, EGF receptor trafficking 54, protein recruitment, and intracellular trafficking 55. Further investigations into the types of SMURF2-mediated ubiquitination may lead to novel strategies for facilitating antitumor immune responses 56.

Previous studies have shown that ubiquitination can regulate both the tumor-promoting and tumor-suppressing pathways in a substrate-specific response 57. Aberrant expression or regulation of E3 ligases is associated with a variety of human tumors by controlling the activity or degradation of tumor-related proteins and has been found in prostate cancer 58-60, colorectal cancer 61, gastric cancer 62, 63, breast cancer 64, 65, esophageal tumor 66, skin cancer 67, hepatocellular carcinoma 68, glioma 69, and NSCLC 70. These E3 ligases exhibit both tumor-promoting and tumor-suppressing functions in a context-dependent manner. Our data delineate a new mechanism to understand SMURF2-mediated ID2 degradation in regulating NSCLC cell proliferation via p21 upregulation.

Nevertheless, this study had some limitations. As important ubiquitin ligases, smurf2 and smurf1 knockout mice exhibit embryonic lethality. We identified the tumor-suppressing role of SMURF2 in LUAD, but whether SMURF1 has the same effect needs to be further studied. According to the results of the interaction between SMURF2 and ID2, it seems that the presence of several domains may reduce the capacity of SMURF2 to bind ID2. More research is needed to understand the potential mechanisms and causes. Furthermore, we detected the increased p21 mRNA levels by overexpressing E2A, but the depletion experiments were lacking. In addition, we propose that ID2 is ubiquitinated and degraded by SMURF2 in NSCLC cells; however, whether SMURF2 targets other proteins during lung cell cycle progression remains to be elucidated. Besides ID2, there are still many other interesting proteins predicted to interact with SMRUF2 and their molecular processes have not been revealed, which deserve further in-depth investigation in the future. Moreover, given the limited *in vivo* data in this study, more animal model-based and clinical studies are required to further consolidate the current findings.

## Conclusions

In the current study, we showed that the E3 ubiquitin ligase SMURF2 plays a role in the cell cycle regulation pathway in NSCLC. SMURF2 directly interacts with ID2, thereby inhibiting the complex formation between ID2 and E2A and preventing transcription of p21. Cancer progression and metastasis consists of several vital steps and is regulated by numerous proteins. ID2 is a multifunctional transcriptional regulator associated with tumor growth and malignant behavior. Therefore, accurate regulation of the intracellular ID2 content is crucial for maintaining normal cell function and developing tumor-targeted drugs. We found that ID2 is a tumor-promoting factor that promotes the progression of NSCLC. Ubiquitination is an important form of PTM that involves the binding of ubiquitin proteins to certain substrates that regulate many human biological processes and tumors. However, more in-depth research is still needed to specify the detailed process of tumor development to find potential or improve existing treatments and ultimately improve the survival time and quality of life of patients with tumors.

## Supplementary Material

Supplementary figures.

## Abbreviations

bHLH: the basic helix-loop-helix
CDKs: cyclin-dependent kinases
ChIP: Chromatin Immunoprecipitation
Co-IP: Co-immunoprecipitation
DMEM: Dulbecco's modified Eagle medium
E2A: transcription factors E12 and E47, encoded by Tcfe2a
FBS: Fetal bovine serum
HECT: homologous to the E6-accessory protein C-terminus
HLH: helix-loop-helix
ID2: inhibitor of DNA binding 2
IHC: Immunohistochemistry
KD: Knockdown
KEGG: Kyoto Encyclopedia of genes and genes
NC: Negative control
NSCLC: non-small cell lung cancer
OE: Overexpression
PBS: Phosphate Buffered Saline
PTM: post-translational modification
RT-qPCR: Real Time Quantitative PCR
SD: Standard deviation
shRNA: Short hairpin RNA
SMAD: Small mothers against decapentaplegic
SMURF2: SMAD specific E3 ubiquitin protein ligase 2
TF: transcription factor
UPS: ubiquitin-proteasome pathway
WB: Western blotting
