# Supplementary material for: SMURF2 facilitates ubiquitin-mediated degradation of ID2 to attenuate lung cancer cell proliferation
Source: Int J Biol Sci. 2023 Jun 26;19(11):3324–40. doi: 10.7150/ijbs.80979 (PMC10367561; doi:10.7150/ijbs.80979)
Supplement: Supplementary file 1 — Supplementary figures. [file ijbsv19p3324s1.pdf]

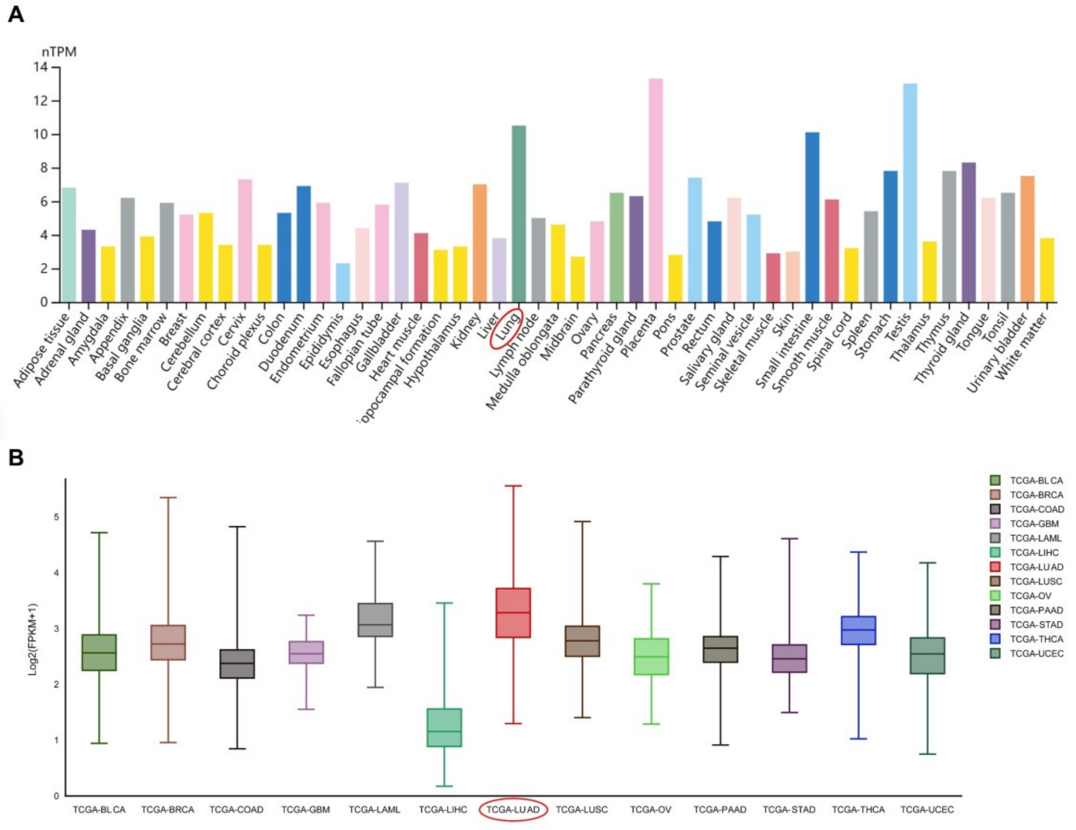

Figure S1. Expression level of SMURF2 in human normal and tumor tissues. (A) HPA dataset was used to evaluate the expression of SMURF2 in human normal tissues. The HPA RNA-seq tissue data was reported as nTPM, corresponding to mean values of the different individual samples from each tissue. Color-coding was based on tissue groups, each consisting of tissues with functional features in common. **(B)** SMURF2 expression in tumors was explored based on TCGA database. The relative expression level was calculated and appeared as Log2(FPKM+1).

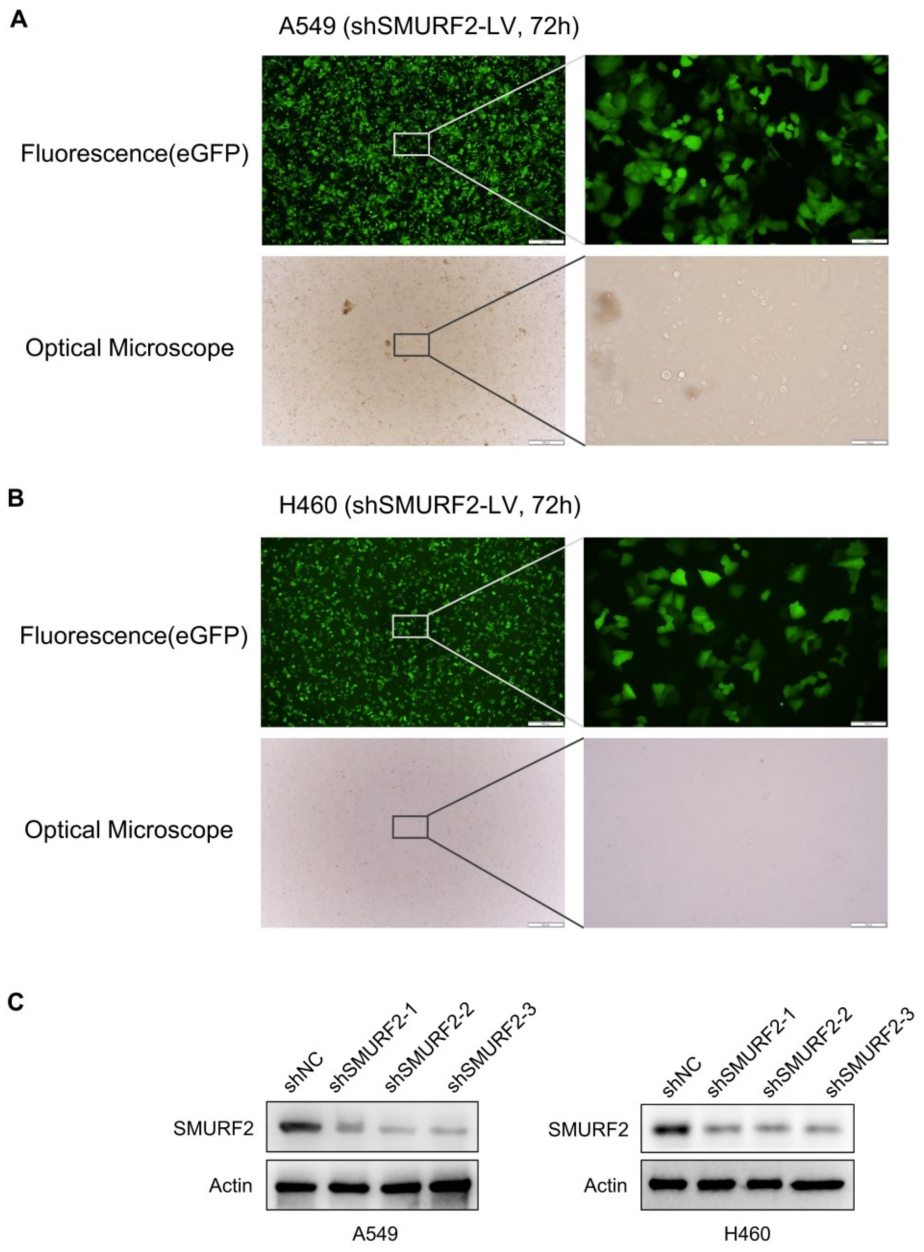

Figure S2. Detection of SMURF2 knocking down efficiency in A549 and H460 cells. **(A)** GFP expression was examined about 72 h post infection by fluorescence microscopy in A549 cells. **(B)** GFP expression was examined about 72 h post infection by fluorescence microscopy in H460 cells. **(C)** The three shRNAs against SMURF2 inhibited the protein expression of SMURF2 in both A549 and H460 cell lines. The scale bars were superimposed on the images (low magnification for 500 μm and high magnification for 100 μm).

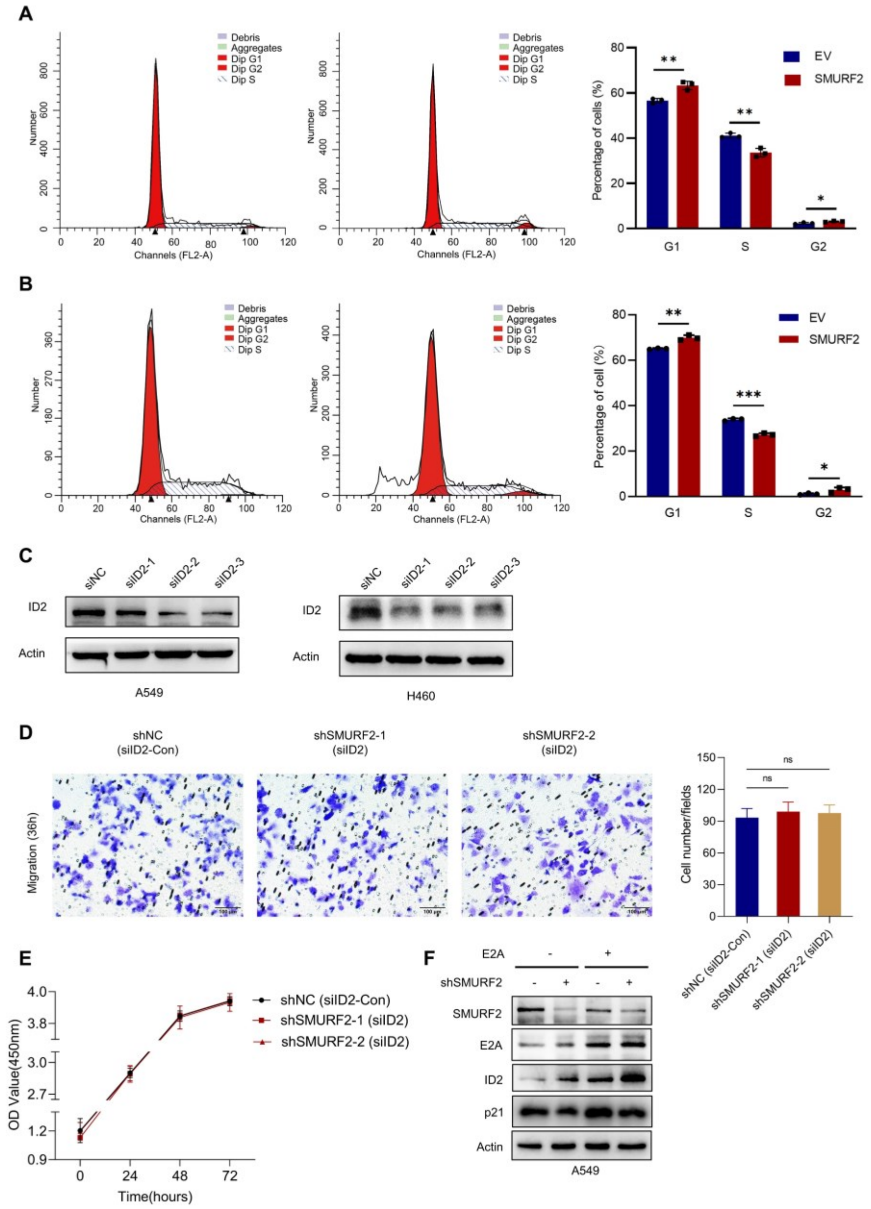

Figure S3. Cell cycle and proliferation were examined following SMURF2 overexpressed in A549 and H460 cells. (A) The cell cycle was detected and further calculated in A549 cells. **(B)** The cell cycle was detected and further calculated in H460 cells. **(C)** Three siRNAs against ID2 inhibited the protein expression of ID2 in both A549 and H460 cell lines. **(D)** The elevated cell migration resulting from SMURF2 knockdown was reversed by the downregulation of ID2. Scale bar, 100 μm. **(E)** The increased OD values following SMURF2 knockdown were restored by the knockdown of ID2. **(F)** Overexpression of E2A restored p21 expression following SMURF2 knockdown. $^{*}P < 0.05$; $^{**}P < 0.01$; $^{***}P < 0.001$; ns, no significance.

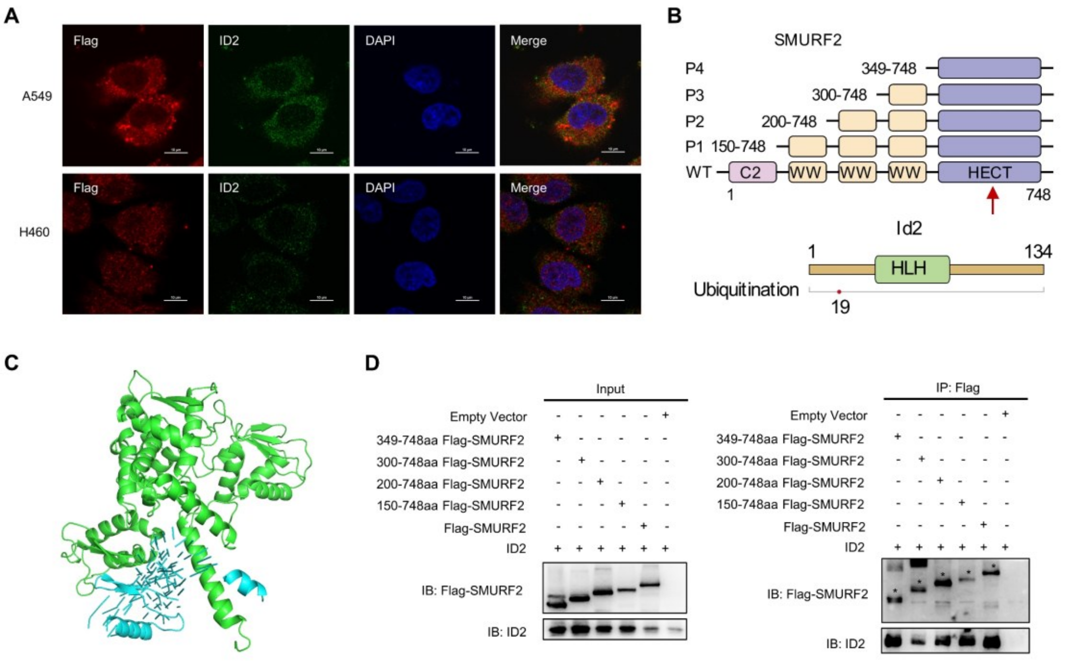

Figure S4. The interaction between SMURF2 and ID2 was explored. (A) Immunofluorescence staining exhibited that exogenous SMURF2 and ID2 were colocalized in A549 and H460 cells. Scale bars, 10 µm. **(B)** Schematic representation of SMURF2-truncated fragments (upper) and ID2 (lower). **(C)** 3D Structure displayed the interaction between SMURF2 and ID2. **(D)** The interaction domain between SMURF2 and ID2 was detected by WB.

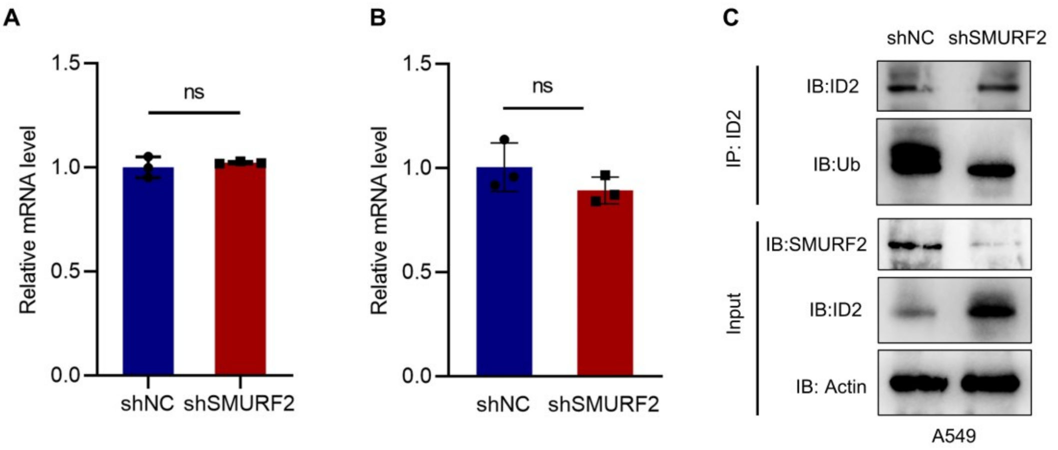

Figure S5. Ubiquitination of ID2 mediated by SMURF2. (A) mRNA expression level of ID2 was detected by RT-qPCR in A549 cells. **(B)** mRNA expression level of ID2 was detected by RT-qPCR in H460 cells. **(C)** Immunoprecipitation with ID2 using SMURF2 knockdown A549 cells revealed a significant decrease in the ubiquitination of ID2. ns, no significance.

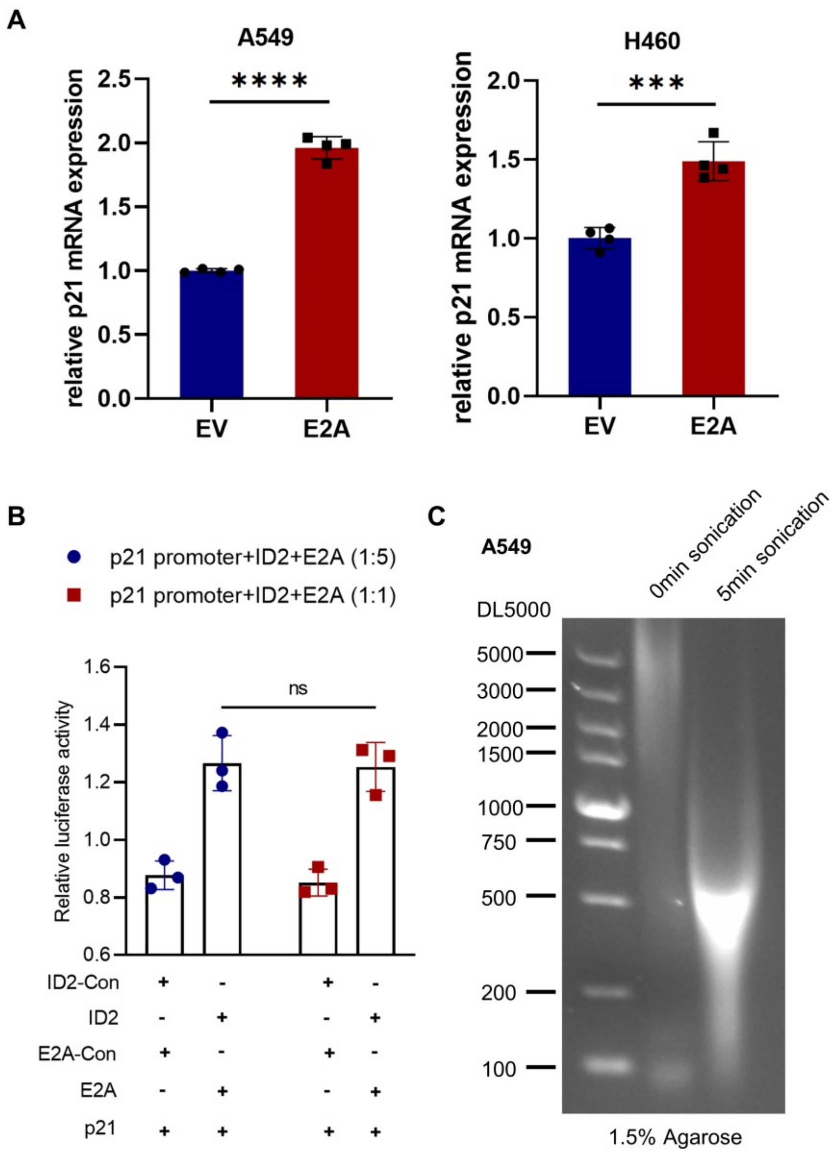

Figure S6. E2A transcriptionally upregulated the expression of CDKN1A. (A) mRNA expression level of p21 was detected in A549 and H460 cells following the overexpression of E2A. **(B)** Relative luciferase activity CDKN1A was detected at different ratios of ID2 and E2A. **(C)** Sheared chromatin from formaldehyde-cross-linked A549 cells was resolved by electrophoresis through a 1.5% agarose gel, with the majority of the DNA had been sheared to a length between 200 bp and 1000 bp. $^{***}P < 0.001$; $^{****}P < 0.0001$; ns, no significance.
